# Profound Rhabdomyolysis and Viral Myositis Due to SARS-CoV-2: A Case Report

**DOI:** 10.7759/cureus.61172

**Published:** 2024-05-27

**Authors:** Makenzie Dye, Rebekah Lantz

**Affiliations:** 1 School of Medicine, Wright State University Boonshoft School of Medicine, Fairborn, USA; 2 Hospital Medicine, Miami Valley Hospital, Dayton, USA

**Keywords:** angiotensin-converting enzyme 2 (ace-2), resuscitation, inflammation, pandemic, elevated ck, fever, myalgias, myoglobinuria, covid-19, rhabdomyolysis

## Abstract

The novel SARS-CoV-2 introduced several new inflammatory conditions including SARS-CoV-2-associated rhabdomyolysis and viral myositis. We present a 22-year-old man who noted a week of cough followed by myalgias, dark-colored urine, and decreased oral intake. He was found to have acute nontraumatic rhabdomyolysis after an acutely positive SARS-CoV-2 test. Initial creatine kinase (CK) level was above the reference range as were liver enzymes reflective of muscle breakdown. Treatment involved fluid resuscitation and pain control, with close monitoring of kidney, liver, and skeletal markers over five days of hospitalization till there was clinical and symptomatic improvement.

## Introduction

In the context of the SARS-CoV-2 era, it is necessary to recognize both the respiratory and extrapulmonary complications of the virus. As many as 2.2% of patients affected with SARS-CoV-2 may have rhabdomyolysis, a condition characterized by the breakdown of skeletal muscle [1] and may be a specific subtype of viral myositis [2]. It is important to recognize this condition due to mortality rates as high as 30% [3]. In the setting of acute kidney injury (AKI), fatality can be as high as 40% [3]. Certainly, rhabdomyolysis seems to be associated with higher risks of decompensation, such as intensive care unit (ICU) admission (90.9%), compared to medical and stepdown comparators (p < 0.001) and mechanical ventilation (86.4%) (p < 0.001) [1].

Risk factors for SARS-CoV-2 rhabdomyolysis include pre-existing health conditions such as advanced age, hypertension, diabetes, cardiovascular disease, and underlying kidney disease [4]. Underlying musculoskeletal disorders and prescription medications such as statins, especially lipophilic varieties, and antipsychotics are thought to increase risk [5]. There may be a direct underlying interaction between the drug and the infection, causing oxidative stress and mitochondrial dysfunction, which impair energy supply, increase demand, and reduce reserves [5]. Dehydration, fever, and respiratory distress can further heighten the body’s immune response, leading to muscle inflammation and damage [6,7]. As research ensues, an understanding of individual cases and their unique risk profiles is essential to effectively assess and manage the risk of SARS-CoV-2 rhabdomyolysis.

We present a case of rhabdomyolysis in a young patient affected with SARS-CoV2 and his course of management and recovery. Our goal is to contribute to the broader discussion surrounding the incidence, risk factors or absence thereof, and adverse outcomes associated with the novel virus.

## Case presentation

A 22-year-old African American man with no past medical history, not on any home prescriptions or supplements, initially presented to the emergency department (ED) with complaints of cough, fever, chills, nausea, and neck pain. Initial vitals were blood pressure of 128/70 mmHg, oxygen saturation (SpO2) of 95%, pulse rate of 97 beats per minute (bpm), temperature of 100.9 degrees Fahrenheit (°F), and respiratory rate (RR) of 18 breaths per minute. The physical exam was unremarkable including for neurologic or musculoskeletal findings. SARS-CoV-2 reverse transcriptase polymerase chain reaction (RT-PCR) test was positive with a cycle threshold (CT) value of 21.5. A chest X-ray (CXR) showed no infiltrates, effusions, or focality. Due to benign assessment, he was deemed safe for discharge to home with return precautions and symptomatic treatment consisting of Tessalon Perles, naproxen, and ondansetron. He was advised to make an appointment with a primary care provider. It does not appear that a creatine kinase (CK) level had been drawn at that time.

A week later, he returned to the ED with a sequela of worsening proximal myalgias despite improvement in fever, chills, cough, and nausea. The patient described constant generalized muscle aches and soreness, most pronounced in the thighs and shoulders. Myalgias did not improve with rest or over-the-counter therapies, including acetaminophen and nonsteroidal anti-inflammatories. He was also concerned about dark-colored urine but was not eating or drinking as much as his normal due to symptoms in the preceding week. He denied any trauma or specific muscle strain and had taken a hiatus from his resistance training. There was no personal or family history of autoimmune, inflammatory, neurologic, or rheumatologic disease. Vitals were stable with a blood pressure of 128/64 mmHg, pulse rate of 77 beats per minute, temperature of 98.5°F, respiratory rate of 18 breaths per minute, and SpO2 of 96% on room air (RA). Physical exam at this time was notable for proximal upper extremity muscle tenderness and hip flexion weakness. He had appropriate range of motion to all major joints, and there were no rashes or skin lesions. Significant lab results included elevated liver enzymes, with aspartate aminotransferase (AST) 1553 U/L (reference: 0-46 U/L) and alanine transaminase (ALT) 327 U/L (reference: 0-60 U/L). CK was 1658 U/L (0-250 U/L). Urinalysis was brown with turbid clarity, 200 mg/dL of protein, large blood, and five granular casts, suggestive of dehydration (Table 1). Urine drug screen was positive for delta-9-tetrahydrocannabinol, and he admitted to occasional use with further questions as all contributors were in consideration. An acute hepatitis panel was negative for hepatitis A IgM, B core IgM, B surface antigen, and hepatitis C antibody. Chest imaging with CXR still showed no cardiopulmonary manifestation of illness. Given a stable renal function, he was administered intravenous (IV) ketorolac every eight hours as needed for pain limited to five doses and bolused 2L IV crystalloids as normal saline for rhabdomyolysis and dehydration. He was subsequently admitted to the hospital for further care.

**Table 1:** Pertinent lab results at the time of hospital admission ALT: Alanine transaminase; AST: aspartate aminotransferase; BUN: blood urea nitrogen; GFR: glomerular filtration rate; HPF, high power field; LPF: low power field; RBCs: red blood cells; THC: delta-9-tetrahydrocannabinol; UA: urinalysis; WBCs: white blood cells

Lab	Patient’s value	Reference value
METABOLIC PANEL		
BUN	13	3-29 mg/dL
Creatinine	1.2	0.5-1.4 mg/dL
Estimated GFR	88	>=60 mL/min/1.73m2
AST	1,553	0-46 U/L
ALT	327	0-60 U/L
Alkaline phosphatase	71	23-144 U/L
Creatinine kinase	1658	0-250 U/L
URINALYSIS		
Color, urine	Brown	Yellow, colorless
Clarity urine	Turbid	Clear
Protein	200	Negative, 10 mg/dl
Blood, UA	Large	Negative
Squamous epithelial cells	1-5	None seen, 1-5 /HPF
Granular casts	1-5	None seen /LPF
URINE DRUG SCREEN		
THC	Delta-9-tetrahydrocannabinol	Undetectable

**Figure 1:**
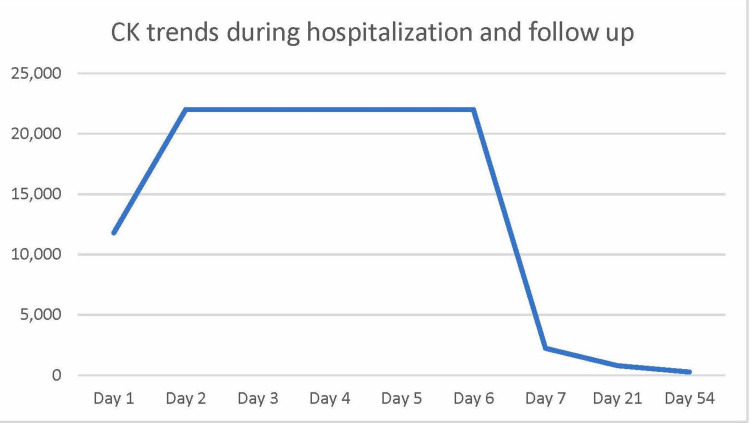
Patient’s chronologic CK trend CK: Creatine kinase The X-axis indicates the day from index hospital admission. The Y-axis indicates the CK level in U/L, where the normal range is 0-250 U/L

**Figure 2:**
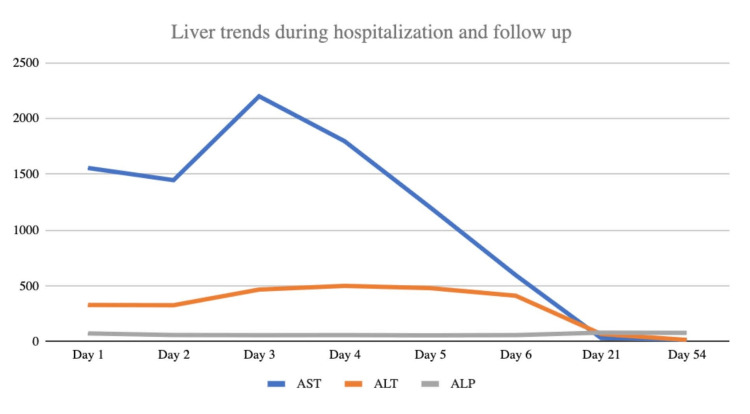
Liver enzyme trends for the patient from hospitalization to follow-up ALP: Alkaline phosphatase; ALT: alanine transaminase; AST: aspartate aminotransferase

CK levels, as a direct measure of skeletal muscle protein leak, were monitored every eight hours. Liver enzymes, especially AST and ALT, were considered an indirect measure of skeletal muscle function and were observed daily. Crystalloids, as IV lactated ringers solution, were administered at an initial rate of 100 cc/hr, which we suspect was the reason for an uptrend in serum markers on days 1 and 2. On day 3, an appropriate resuscitation rate of 250 cc/hr was assumed. CK levels remained elevated for five days before finally down trending (Figure 1), and there was an associated improvement in proximal extremity myalgias. AST and ALT were also noted to be responsive to resuscitation rate as they finally down trended after an increase in the fluid rate (Figure 2). After symptoms and lab markers were markedly improved, he was discharged after a week of hospitalization, in stable condition to home. He was instructed to make an appointment with his primary care office in 1-2 weeks with plan for repeat CK and comprehensive metabolic panel. He was instructed to limit nonsteroidal anti-inflammatory drugs (NSAIDs) to 4 g a day to prevent renal injury. For safekeeping and not to influence liver labs or potential hepatic clearance, he was advised to not take any acetaminophen products in the meantime. He did not require or request any new prescriptions.

At a two-week follow-up on day 21, CK level had improved to 792 U/L, and liver markers had normalized (Figures 1-2). Importantly, he maintained full strength and had resolution of all symptoms.

## Discussion

Acute viral myositis, rhabdomyolysis, dermatomyositis, paraspinal myositis, and myasthenia are the most common musculoskeletal presentations of SARS-CoV-2 [8]. More rarely, axonal neuropathy or cachexia may occur. The manifestation of proximal myalgias in our patient suggests a viral myositis [2]. If rashes had been present, for example, a dermatomyositis would be suspected [4]. Rhabdomyolysis with SARS-CoV-2 is associated with higher rates of ICU admission, mechanical ventilation, and mortality [1,3]. Persistent fatigue and myalgias have been reported as the long-term debilitating symptoms of the condition [8], which our patient fortunately did not endure and had a good clinical response likely due to young age and absence of comorbid risk factors.

It is postulated that SARS-CoV-2 gains direct entry into the muscle tissue via the angiotensin-converting enzyme 2 (ACE-2) receptor, which is present in vascular endothelium, bowel, synovium, and smooth and skeletal muscle. There is an ongoing need for research to better understand the heterogeneity of this complication across different patient populations and geographies [1,3].

Several cases of SARS-CoV-2 viral myositis have been documented in the literature. Our case is added to the collection of Saud et al.'s 22 compiled cases [8], as well as to Jin [6], Chedid [9], and Mukherjee [10] (Table 2).

**Table 2:** Twenty-six cases of SARS-CoV-2 viral myositis Adapted from Saud et al. [8] by coauthor Rebekah Lantz CK: Creatine kinase; CKD: chronic kidney disease; DM: diabetes mellitus; HTN: hypertension; F: female; M: male; NA: not any indicating absence of case report information on this detail

Reference	Demographics	Treatment	Lab trends	Outcomes
Current case	22M	Fluids, toradol	CK 1658 U/L, peak >22,000	Discharged after 14 days of hospitalization
[6]	60M	Fluids, supplemental oxygen, alkalinization, moxifloxacin, interferon nebulizers, opinavir, plasma transfusion, gamma globulin	CK 11,842 U/L, peak 11,842	Discharged after 12 days of hospitalization
[9]	51M, HTN, DM, CKD	Fluids, supplemental oxygen, hydroxychloroquine, remdesivir, tocilizumab, bicarbonate, hemodialysis	CK 339,500 U/L, peak 339,500	Discharged after 15 days of hospitalization
[10]	49M, HTN, DM	Fluids, hydroxychloroquine	CK 22,740 U/L, peak 22,740	Discharged after 8 days of hospitalization
[11]	38M, obesity	Azithromycin, supplemental oxygen, nebulizers, plaquenil, tocilizumab, heparin	CK 588 U/L, peak 33,000	Hospitalized 3 months. At time of the paper was still admitted and pending safe disposition
[12]	67M, HTN	Fluids, azithromycin, ceftrixone, hydroxychloroquine, hemodialysis	CK 586 U/L, peak 19,773	Died on day 21 of hospitalization
[12]	39M, HTN	None	CK 4330 U/L, peak 4330	Died on first day of hospitalization
[12]	43M, CKD	Azithromycin, ceftriaxone, hydroxychloroquine	CK 8636 U/L, peak 9793	Died on day 2 of hospitalization
[12]	70M	Fluids, IV methylprednisolone, ceftriaxone	CK 5008 U/L, peak 5008	Died on day 17 of hospitalization
[13]	78M, DM, HTN	Fluids, hydroxychloroquine, ritonavir, lopinavir	CK 22,511 U/L, peak 22,511	Discharged after 6 days of hospitalization
[14]	38M, DM, gout, obesity	Fluids, albumin, furosemide, potassium, spironolactone, mannitol	NA	Discharged after 23 days of hospitalization
[15]	38M	Fluids, azithromycin → doxycycline, cefepime, hydroxychloroquine	CK 42,670 U/L, peak 42,670	Discharged after 9 days of hospitalization
[15]	58F	Methylprednisolone, azithromycin, hydroxychloroquine, tocilizumab	CK 700 U/L, peak 700	NA
[16]	NA	Fluids	CK 25,384 U/L, peak 25,384	Last in critical condition
[17]	64M	IV antibiotic, prednisolone, hydroxychloroquine, ivermectin, IVIG, mycophenolate mofetil	CK 990 U/L, peak 990	Discharged after 14 days of hospitalization
[17]	50F	Methylprednisolone, cyclophosphamide, methotrexate	CK 150 U/L, peak 150	Demise
[17]	26F	Prednisolone, hydroxychloroquine, methotrexate	CK 8439 U/L, peak 8439	Recovered
[17]	46M	Hydroxychloroquine, methotrexate, mycophenolate mofetil	CK 570 U/L, peak 570	Recovered
[18]	33F	NA	NA	Discharged after 25 days of hospitalization
[18]	60M	NA	NA	Discharged
[18]	63M	NA	NA	Discharged after 64 days of hospitalization
[18]	87M	NA	NA	Discharged
[18]	54F	NA	NA	Discharged after 34 days of hospitalization
[18]	62M	NA	NA	Discharged after 38 days of hospitalization
[18]	56M	NA	NA	Discharged after 30 days of hospitalization
[19]	33M, DM	NA	NA	Bilateral amputation. Discharged post op day 16

These cases contribute to our understanding of an important causative viral myositis. There were varied demographic risk factors, symptomatology, interventions applied, and response to therapies, especially during the peak of SARS-CoV-2-positive hospitalizations from December 2020 to January 2021 [20].

Best practices for managing SARS-CoV-2 myositis and associated rhabdomyolysis involve a comprehensive history from the onset, and workup involving CK level, which were lacking from our patient's primary ED presentation. Early recognition of signs such as persistent myalgias, dark-colored urine, and elevated CK levels is essential [21]. These are the traditional triad of rhabdomyolysis in 10% of patients, although up to 50% of patients present asymptomatically [22]. Regular monitoring of CK levels should definitively be obtained in symptomatic patients with persistent myalgias who have tested positive for SARS-CoV-2, as this action can help identify early elevations indicative of impending rhabdomyolysis [22].

Aggressive IV fluid resuscitation with several liter boluses followed by resuscitative fluids is a cornerstone of management to prevent AKI by promoting myoglobin clearance and maintaining renal perfusion [23]. Symptomatic management for pain involves conservative therapies such as muscle relaxants and general avoidance of nephrotoxins and acetaminophen so as not to influence important lab trends that are important to honor. Our patient did not present with AKI and maintained successful UOP throughout his heavy fluid resuscitation, especially during the five days of 250 cc/hr, so that it is minimally okay to consider NSAID for conservative management. Alternatives such as acetaminophen affect levels of AST and ALT, which are monitored units, and opiates have been seen to contribute to rhabdomyolysis also. As seen, management of viral myositis and rhabdomyolysis can be intricate and an art based on the patient's unique presentation, background factors, and concerns. Involving nephrology early in the patient’s course for comanagement essentially improves patient outcomes [22]. Additionally, after the patient has improved objectively and clinically and been discharged, follow-up care with labs and clinical evaluation during the post-acute phase are important to assess for lingering effects of rhabdomyolysis and kidney function [23].

Further research in the realm of SARS-CoV-2 myositis and rhabdomyolysis should explore the cellular mechanisms of illness and nuances of risk factors, investigate the impact of different treatment modalities, and elucidate the long-term consequences of rhabdomyolysis in SARS-CoV-2 survivors and nonsurvivors [1,24,25,26]. Establishing a best early detection and guideline for therapy can lead to early interventions and prevent downstream complications and mortality.

## Conclusions

SARS-CoV-2 myositis and rhabdomyolysis are important sequelae of the novel virus. They are associated diagnoses that should be recognized early and involve a careful history at initial presentation to divulge the risk factors for severity and decompensation. Prompt aggressive IV crystalloid resuscitation should ensue with strict return precautions for worsening symptoms and consideration for hospitalization. Recognizing the similarities and differences among published cases improves our understanding and management of how to improve our medical care and patient outcomes. Ongoing research should involve early detection methods, refined guidelines, and alternate nuanced options for complex presentations.
